# Comparative Analysis of Protein Extraction Protocols for Olive Leaf Proteomics: Insights into Differential Protein Abundance and Isoelectric Point Distribution

**DOI:** 10.1021/acsagscitech.4c00642

**Published:** 2025-03-13

**Authors:** Bihter Uçar, Merve Öztuğ, Mahmut Tör, Nurçin Çelik-Öztürk, Filiz Vardar, Birsen Cevher-Keskin

**Affiliations:** † Plant Biotechnology Laboratory, The Scientific and Technological Research Council of Turkey (TUBITAK); Marmara Research Center, P.O. Box 21, Gebze 41470, Kocaeli, Turkey; ‡ Department of General Biology, Faculty of Arts and Sciences, Marmara University, Istanbul 34722, Turkey; § National Metrology Institute, Chemistry Group, Bioanalysis Laboratory, The Scientific and Technological Research Council of Turkey (TUBITAK), P.O. Box 21, Gebze 41470, Kocaeli, Turkey; ∥ Department of Molecular Biology and Genetics, Faculty of Science and Letters, Istanbul Technical University, Maslak 34469, İstanbul, Turkey; ⊥ Department of Biological Sciences, School of Science and the Environment, University of Worcester, Worcester WR2 6AJ, U.K.

**Keywords:** Olea europaea, proteomic, protein extraction protocols, LC–MS/MS, CHAPS, SDS, TCA/acetone

## Abstract

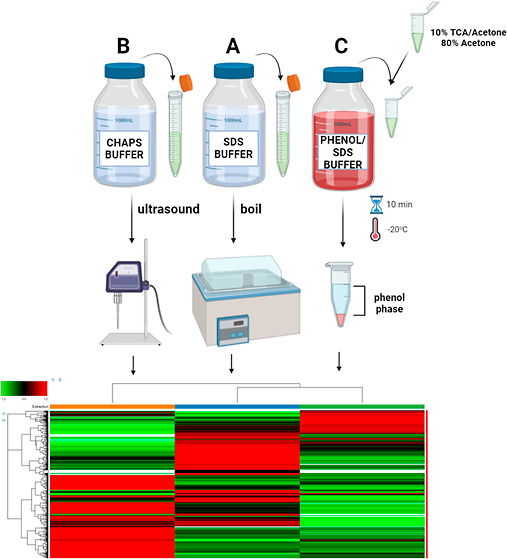

Plant proteomics studies face two major challenges: limited databases due to the need for sequenced genomes and the difficulty in obtaining high-quality protein extracts. Olive (*Olea europaea*), a key species in Mediterranean flora known for its rich biochemical content, presents additional complexity due to its lipidic structure and high levels of inhibitory compounds that hinder protein extraction. Consequently, various studies have focused on optimizing the protein extraction methods for olives. While different extraction protocols exist for leaf proteome analysis, their compatibility with LC–MS/MS has been scarcely studied. This work was carried out to compare three protein extraction protocols for LC–MS/MS analysis using olive (*O. europaea* L) leaf tissue. Denaturing SDS (Method A), physiological CHAPS (Method B), and phenolic TCA/acetone (Method C) were evaluated with LC–MS/MS data. The quantitative comparisons of the three extraction methods revealed that Protocol A gave the greatest yields. According to the results obtained, Protocol A uniquely identified 77 proteins, Protocol B identified 10 unique proteins, and Protocol C identified 19 unique proteins. Similarly, the peptide sequence analysis showed that Protocol A uniquely identified 208 peptide sequences, Protocol B identified 29, and Protocol C identified 36. Moreover, reversed-phase high-performance liquid chromatography (RP-HPLC) results suggest that Method A may be more efficient in removing and retaining hydrophobic proteins. Overall, Protocol A demonstrated greater sensitivity, efficiency, and reproducibility in LC–MS/MS analysis.

## Introduction

The olive tree is a strategic plant with a wide genetic heritage that includes more than 1200 varieties as well as many wild varieties.^1^ Adaptation to different abiotic stresses over centuries has enabled such a great genetic diversity. With the increasing requirement for table olives and olive oil with high quality standards, the characterization of olive genetic resources has become an important issue. Agronomic, morphological, and biochemical characteristics have been widely applied to distinguish between olive varieties, determine the origin of varieties, and also to study genetic diversity.^2−4^

The improvement of DNA molecular techniques has been accelerated by the development of effective genetic markers for the characterization of olive varieties.^5^ For the identification of olive varieties, SSR markers were able to discriminate varieties and are considered a suitable technique. However, SSR markers are limited for appropriate identification due to the low number of polymorphisms.

“Chemometric approaches” based on analytical data have recently gained importance for the characterization of olive varieties. Analyses were accomplished on olive tissues such as leaf or olive fruit.^1,6^ There are studies in which the differences between olive tree varieties in the same geographical region are determined depending on chemotaxonomic markers, such as secondary metabolites. The studies were carried out as a result of detailed examination of the phenolic profiles obtained by chemometric LC–MS/MS that allowed discrimination between various types of olives.^1^

Proteomics is defined as the in-depth investigation of different proteins expressed by the organism.^7^ Its purpose is to characterize as many proteins as possible structurally, physico-chemically, and biologically.^8^ Proteomic studies conducted for this purpose allow simultaneous examination of the total proteome, its quantitative abundance and qualitative presence, diversity, and localization within a population.^7^ Recently, the field of proteomics has evolved toward more functional approaches whose priority is aimed at elucidating molecular mechanisms that regulate cellular activities rather than identifying protein or post-translational modifications.^9^

Proteomic include techniques based on mass spectrometry (MS) as the core platform.^8^ Quantitative proteomics using liquid chromatography (LC)-tandem mass spectrometry (MS/MS) represents the preferred cutting-edge technological approach for proteome characterization and quantitative analysis of the diversity of post-translational modifications.^9^ Since protein synthesis and regulation are directly altered by many environmental factors, this method is necessary to effectively identify plant phenotypic changes, especially against biotic and abiotic environmental stresses.^10^ Genomic and transcriptomic studies, which are widely used today, cannot monitor post-transcriptional processes, leading to increasing interest in proteomics in comprehensive studies.^11^ The liquid chromatography–mass spectrometry (LC–MS) technology has become the ideal method for the localization and subcellular level dynamics of proteins. The capability of measuring the complex protein mixtures has made it one of the recently used methods.^12^ It can show the quantitative state of a proteome and support the resolution of the cellular signaling networks and protein–protein interactions. This technology is also useful in elucidating the molecular mechanisms of different biotic and abiotic stress responses in plants.^13^

Nonetheless, proteomic analyses are also challenging due to the significant complexity of the proteome, which includes various post-translational modifications, different protein interactions, and complex features such as protein folding.^14^ In addition, the limited database because of the lack of sequenced plant genomes is one of the biggest problems encountered when working with plant proteins. The accuracy and efficiency of the proteomic study depend on the effective choice of methods used in the protein analysis steps (isolation, digestion, separation, identification, and quantification of proteins).^7^ Protein extraction is one of the most critical steps in the proteomics workflow.^15^ In particular, compounds contained in plant tissues that affect the advanced stages of protein analysis, such as phenolics, organic acids, pigments, and polysaccharides, prevent the production of high-quality samples.^9,16^ In recent years, new methods have begun to be developed to improve and streamline the sample preparation process and integrate sample preparation and fractionation. In this way, it has been reported that experimental time is reduced, efficiency is increased, and repeatability is improved.^15^

*Olea europaea* L. has significant effects on health owing to its richness in a wide range of phenolic compounds such as flavonoids, secoiridoids, tyrosol, caffeic acid, and ferulic acid.^17^ Although *O. europaea* has a rich compound content, it faces a lack of proteomic research.^18^ Proteins in olives have yet to be investigated in detail, probably due to the complexity of working with such phenolic and lipid compounds and, therefore, it is tough to detect the amount of protein in this biological material. One of the most important reasons is that the olive plant contains phenolic and lipid compounds that lead to low-quality extracts, reducing protein extraction efficiency.^10^

Protein extraction from olive leaf tissues has several difficulties because of the low level of protein content and the presence of high levels of polyphenols (flavonoids, phenolic acids, etc.) and secondary metabolites.^19^ Binding of polyphenolic compounds to proteins interferes with protein extraction, causing precipitation and inhibiting enzymatic activity.

To increase the amount of protein extraction, optimization of extraction protocols and the use of appropriate protein quantification assays are important. Olive leaf tissues include endogenous enzymes that can degrade proteins during extraction.^17^ The addition of protease inhibitors to extraction buffers prevents enzymatic degradation and preserves protein integrity. The protein content of olive leaves may vary depending on factors such as variety, age of the leaf, and environmental conditions.^17^ To accomplish consistent and reproducible protein extraction between different samples, detailed standardization of the extraction protocols is required. In spite of these difficulties, with careful optimization of extraction methods and the use of suitable buffers and inhibitors, it is possible to effectively extract proteins from olive leaves for different downstream applications.

The dynamic range of protein concentrations in olive leaf extracts can fluctuate broadly, with some proteins present in high abundance, while others are low in abundance. Accurate quantification of this range requires the careful optimization of instrument parameters and data acquisition methods. Proteins in olive leaf extracts are prone to degradation during sample preparation, storage, or analysis, causing the generation of nonspecific peptides or loss of protein coverage.^9^ For that reason, appropriate sample handling and storage are crucial to obtaining reliable LC–MS/MS data. LC–MS/MS data analysis of olive leaf extracts comprises complex data processing and interpretation steps. Database searching, peptide identification, and protein quantification steps require the correct bioinformatics tools to obtain meaningful biological understanding from the data.

The primary purpose of this study is to develop an effective protein extraction method that can identify the highest protein content in the olive plant with high efficiency for LC–MS/MS. Studies in this field are also important in terms of the characterization of olive proteins, the discovery of new bioactive molecules, and understanding the value of olive products. For this purpose, three protein extraction protocols were tested, and LC–MS/MS data were compared in olive plant leaf tissues.

## Experimental Design

### Sample Preparation

#### Plant Material

Olive leaves were collected from TUBITAK Gebze Campus (Kocaeli-Turkey, 40°47′08″N 29°26′57″E). Freshly collected leaves were frozen in liquid nitrogen and stored at −80 °C until protein extraction.

### Protein Extraction

**Figure 1:**
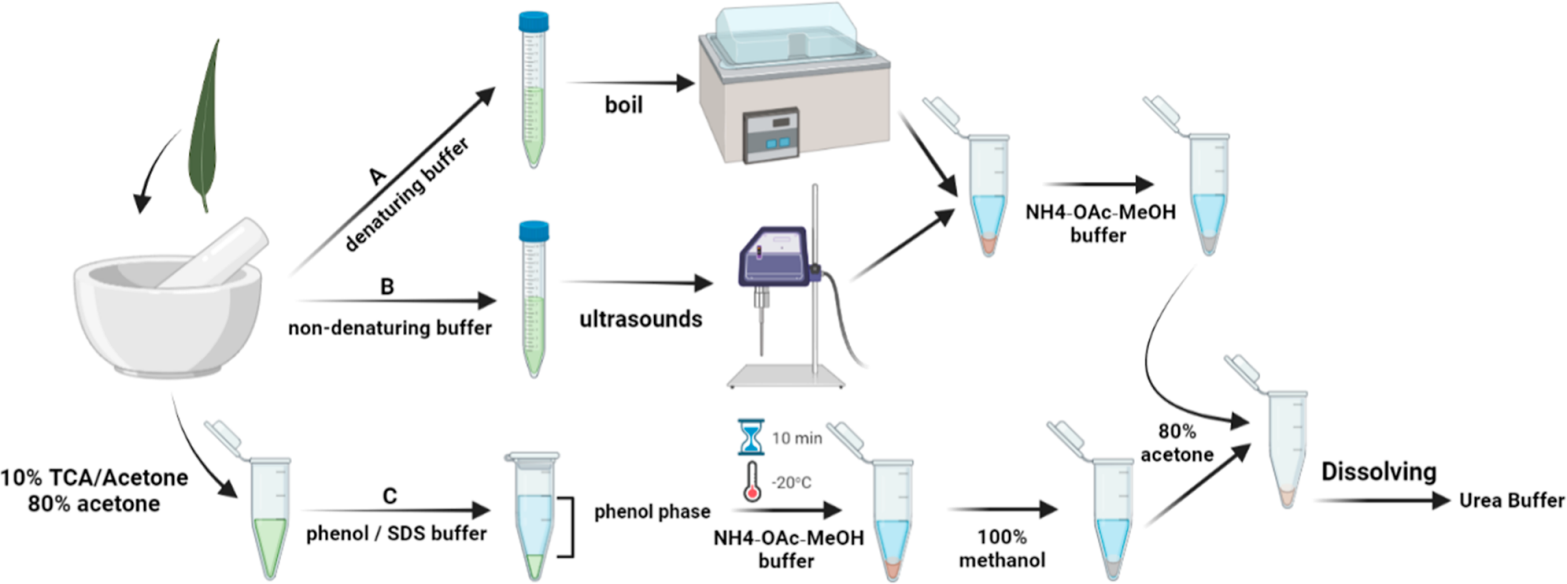
Protein extraction and precipitation methods for olive leaf: (A) Protocol A—SDS, (B) Protocol B—CHAPS, and (C) Protocol C—Phenol/SDS.

Three extraction protocols were carried out in three different conditions: denaturing (extraction A, SDS), physiological (extraction B, CHAPS), and phenolic (extraction C, TCA/acetone) conditions.^20−24^ First, in all extraction protocols, the olive leaves were ground to a fine powder with liquid nitrogen using a pestle and mortar. For extraction A and B, olive proteins were extracted as described by Capriotti et al. (2013) with the following adaptations applied.^24^ For both extractions (A and B), the same precipitation protocols were employed. The last extraction protocol (extraction C, TCA/acetone), which was performed using phenolic conditions, differed from those of the other two. For extraction C, based on the work of Wang et al. (2006), the procedure was performed by adapting the TCA/acetone combination, methanol washes and a phenol extraction.^20^Figure 1 shows the methods of all protein extraction and precipitation. The Qubit Protein Assay Kit (Invitrogen) was used for the determination of the protein concentrations, which is used as the gold standard for LC–MS/MS studies.

## Chemicals, Biologicals, and Apparatuses

Denaturing extraction buffer [0.125 mol L^–1^ tris-HCl (pH 7.4), 5% (v/v) glycerol, 3% (m/v) SDS, 1% (v/v) protease inhibitor cocktail, and 25 mmol L^–1^ DTT], physiological (nondenaturing) extraction buffer [0.125 mol L^–1^ tris-HCl (pH 7.4), 50 mmol L^–1^ NaCl, 3% (m/v) CHAPS and 1% (v/v) protease inhibitor cocktail], phenolic extraction buffer [1:1 phenol (tris-buffered, pH 8.0)/SDS buffer (30% sucrose, 2% SDS, 0.1 M tris-HCl pH 8.0) and β-mercaptoethanol (15 μL β-mercaptoethanol for each 1.5 mL SDS buffer)], ice-cold 80% methanol containing 0.1 M ammonium acetate (NH_4_-OAc-MeOH), ice-cold 80% acetone, urea buffer [50 mM tris-HCl pH 6.8, 0.2% SDS, 8 M urea and β-mercaptoethanol (10 μL β-mercaptoethanol for each 1 mL SDS buffer)], 10% TCA/acetone buffer, and 100% methanol were used for all extraction and precipitation steps of olive proteins.

## Method A (Denaturing, SDS)

For 1 g of ground leaf tissue, 5 mL of a denaturing extraction buffer was used. The resulting suspension was vortexed for a few minutes and then boiled for 10 min in a 15 mL tube. The insoluble fraction of extract was removed by centrifugation at 12,300*g* for 10 min (RT). The mixture, containing the total proteins, was transferred into a new 2.0 mL Eppendorf tube and mixed with three volumes of ice-cold 0.1 M ammonium acetate in methanol buffer. Protein pellet was obtained by centrifugation at 21,000*g* for 5 min (4 °C) and washed twice in NH_4_-OAc-MeOH buffer. The proteins were precipitated again twice in ice-cold 80% acetone buffer. The resulting suspension was vortexed for a few minutes. The final pellet was dissolved in 150 μL of urea buffer as described by Maayan et al. (2008).^25^

## Method B (Physiological, CHAPS)

1 g of ground leaf was homogenized with 3 mL of a nondenaturing buffer. The mixture thus obtained was subjected to three cycles of ultrasounds, for 30 s at 30% amplitude, using an ultrasonic microprobe (Branson-SFX150) into a 15 mL tube. The insoluble matter was removed by centrifugation at 12,300*g* for 15 min (RT). The mixture, containing the total proteins, was transferred into a new 2.0 mL tube and mixed with three volumes of ice-cold 0.1 M ammonium acetate in methanol buffer. The proteins were pelleted by centrifugation at approximately 21,000*g* for 5 min (4 °C). The protein pellet was washed twice in NH_4_-OAc-MeOH buffer and precipitated again twice in ice-cold 80% acetone buffer. The final pellet dissolved in 100 μL urea buffer as described by Maayan et al. (2008).^25^

### Method C (Phenolic, TCA/Acetone)

The leaf powder (0.2 g) was transferred to a 2 mL tube and mixed with 0.5 mL of 10% TCA/acetone. The resulting suspension was vortexed for a few minutes and then pelleted by centrifugation at approximately 16,000*g* for 10 min (4 °C). The supernatant was removed by careful pipetting. The pellet, containing the total proteins, was washed once with 80% methanol containing 0.1 M ammonium acetate and once with 80% acetone. During each wash step, the pellet was mixed by vortexing for a few minutes and then centrifuged at approximately 16,000*g* for 10 min (4 °C). After the washing steps, the supernatant was removed by careful pipetting. The pellet was incubated for at least 10 min at 50 °C to remove residual acetone. Next, 2 g of the dry powder of leaf tissue was resuspended in 1.8 mL of 1:1 phenol/SDS buffer in a 2.0 mL tube. The mixture thus obtained was mixed thoroughly and was incubated at 95 °C for 5 min. The mixtures were centrifuged for 5 min at approximately 16,000*g*. The upper phenol phase, containing the total proteins, was transferred into a new 2.0 mL tube, mixed with five volumes of cold 80% methanol containing 0.1 M ammonium acetate, and stored at −20 °C overnight. The mixtures were pelleted by centrifugation at approximately 16,000*g* for 10 min (4 °C). The supernatant was discarded carefully when the pellet looked white. The protein pellets were washed once with 100% methanol and once with 80% acetone. During each wash step, the pellet was mixed by vortexing and then centrifuged. The final pellet was dissolved in 100 μL urea buffer as described by Maayan et al. (2008).^25^ For each of the three extraction protocols, three experimental replicates were performed.

### Preparation of Tryptic Peptides

Peptides were obtained using the FASP Protein Digestion Kit (Expedeon) according to the manufacturer’s protocol. Protein concentration was determined by Qubit assay, and a 25 μg sample was transferred to the 30 kDa cutoff columns. Denaturation, alkylation, and washing steps were performed exactly according to the protocol. Next, 50 μL of trypsin solution [20 μg of trypsin (Promega) in 1000 μL of 50 mM ammonium bicarbonate] was added to the columns (protein/enzyme ratio of 25:1) and incubated at 37 °C overnight (∼16–18 h). The solution on top of the filter, containing the peptide digest, was transferred to clean Eppendorf tubes, and 40 μL of 50 mM ammonium bicarbonate was added to the sample and centrifuged at 14,000*g* for 10 min. This step was repeated twice. Subsequently, 50 μL of 0.5 M NaCl solution was added and centrifuged at 14,000*g* for 10 min. Peptides passing into the lower tube were taken into Protein-LoBind tubes, and the peptide concentration was measured with NanoDrop. The samples were diluted with 0.1% formic acid to a final concentration of 250 ng/μL and transferred to the appropriate tubes for the device.

### LC–MS/MS Analysis for Protein Identification and Quantification

The analysis was conducted using a Thermo Scientific UltiMate 3000 RSLC Ultra Nano ultraperformance liquid chromatography (UPLC) system paired with a Thermo Scientific Q Exactive HF mass spectrometer equipped with a Thermo Scientific EasySpray ion source.

Peptides were first retained in the trap column (C18 PepMap100, 5 μm, 100A, 300 μm i.d. × 5 mm) and subsequently separated on an analytical column (C18 Easy-Spray ES804A RSLC C18, 2 μm, 100A 75 μm × 15 cm) using an acetonitrile gradient created by the nanopump. Peptides were separated over 50 min at a flow rate of 300 nL/min in the analytical column, maintained at 40 °C. 500 ng of peptide was loaded onto a 150 mm Nano LC column. Mobile Phase A consisted of 0.1%FA in 98:2% H_2_O, and Mobile phase B consisted of 0.1% FA in 98:2% acetonitrile. The gradient time was programmed as follows: phase B increased from 3% to 8% in 0.09 min, from 8% to 24% in 0.76× min, and from 24% to 36% in 0.15× min. This was followed by an increase to 64% B within 0.5 min and maintained at 64% B for 6.5 min. The flow rate was kept at 300 nL/min. Re-equilibration was achieved by washing with a 3% B solution for 10 min.

The Q Exactive HF-X instrument (Thermo Fisher Scientific, Bremen, Germany) was calibrated by using the instrument control software Tune (Version 2.9). The spray voltage was set at 2 kV, the funnel RF level was 50, and the capillary temperature was 270 °C. For data-dependent acquisition (DDA) analysis, the device was configured in “Full MS/DD–MS/MS” mode. Full MS resolution was set to 120,000 for *m*/*z* 200, with a Full MS AGC target of 3E6 and an injection time (IT) of 100 ms. The mass range is defined as 350–1400. The MS/MS AGC target value is set to 5 × 10^4^, “The intensity threshold” is set to 4 × 10^4^ and the isolation width is 1.2 *m*/*z*. The normalization collision energy is set to 28%. All data were obtained in the positive ion mode.

Extraction and analysis of mass data was achieved using TraceFinder Software. Data analysis was performed using Proteome Discoverer 2.4 software with the Sequest HT search algorithm. The files were searched against the NCBI Viridiplantae (Taxonomy No: 33,090) database using the Sequest HT search engine with a Strict FDR of 0.01. Preliminary mass tolerance of 10 ppm and fragment mass tolerance of 0.02 Da were used as the Sequest HT parameters.

Quantification of proteins was performed using the label-free quantification (LFQ) algorithm within Proteome Discoverer 2.4. Significant differences in protein abundances, with a greater than 2-fold change (log2 FC: 1 and −1), were assessed using Proteome Discoverer false discovery rate-adjusted *P*-values (*P* < 0.05).

## Results and Discussion

Proteomics has become an indispensable tool for understanding plant biology at the molecular level, enabling the identification of proteins involved in metabolism as well as abiotic and biotic stress responses. For example, 2D-SDS PAGE analysis has been employed to identify differentially expressed proteins in jojoba leaves contributing to biomarker development.^26^ Similarly, proteomic studies of *Cycas revoluta* (sago palm) have revealed stress tolerance mechanisms by identifying proteins associated with environmental adaptations.^27^ These methodologies underscore the transformative impact of proteomics in plant research, offering valuable insights into complex biological processes.

**Figure 2:**
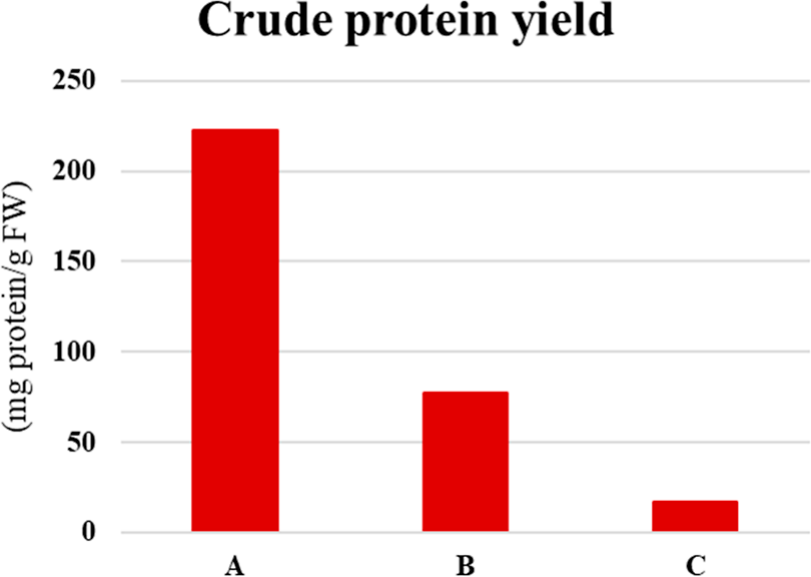
Comparison of crude protein yields for three extraction methods of olive leaf. FW = fresh weight.

This study aims to compare three different protein extraction methods (Figure 1) with LC–MS/MS data and evaluate the effectiveness of the methods, determining protein quantities and quality for each method. The quantitative comparisons of the three extraction methods revealed that Protocol A gave the maximum yield among the three methods (Figure 2).

**Figure 3:**
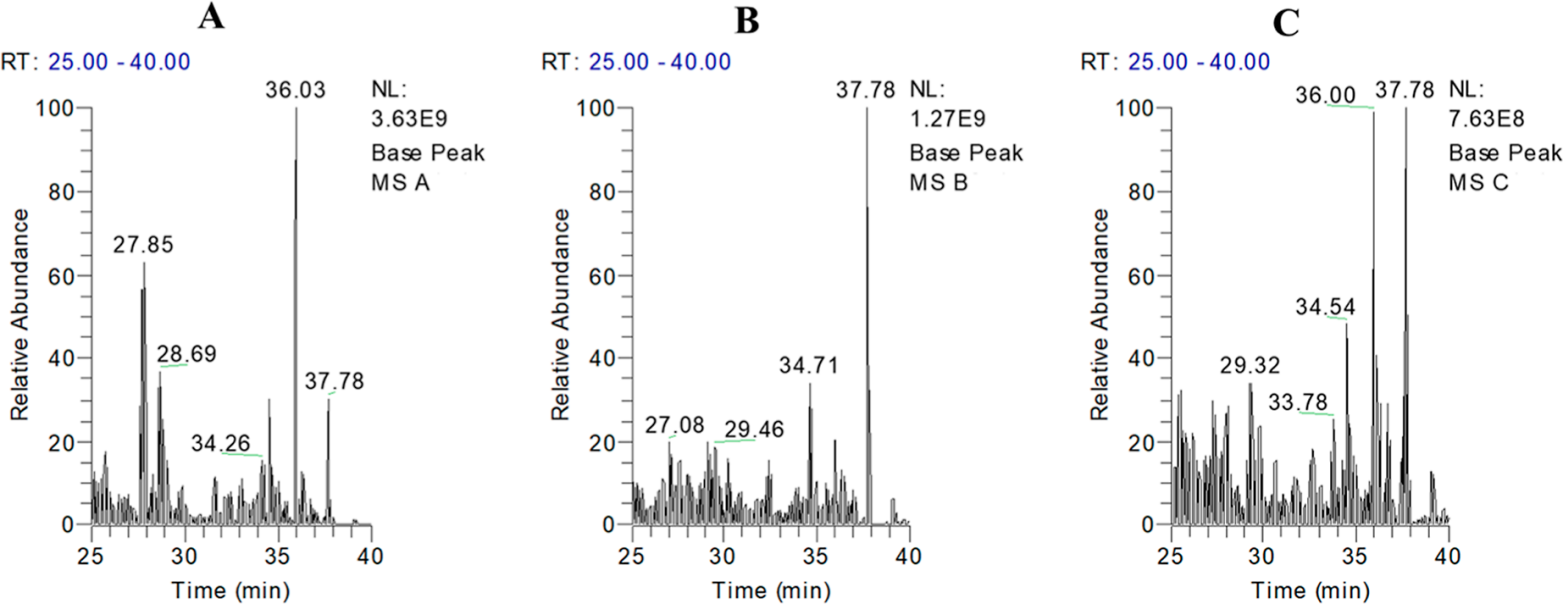
LC–MS base peak chromatograms of peptides obtained using extraction protocols: (A) Protocol A, (B) Protocol B, and (C) Protocol C. Retention time 25–40 min. Highlighting hydrophobic peptides.

Following the extraction and subsequent trypsin digestion of proteins using the filter-assisted sample preparation (FASP) method, the resulting peptides were subjected to LC–MS/MS analysis using the same gradient for all three extraction protocols. Data analysis was performed by using Proteome Discoverer 2.4 software with the Sequest HT search algorithm. Files were identified using the Sequest HT search engine with an FDR of 0.01 in the NCBI Viridiplantae (Taxonomy Number: 33,090) database. A precursor mass tolerance of 10 ppm and a fragment mass tolerance of 0.02 Da were used as Sequest HT parameters. As a result of Sequest HT screening, 2259 protein groups, 5751 peptide groups, 74,797 PSMs, and 566/6315 annotation protein groups were detected. Each protein sample was analyzed three times, and peptides identified in at least two analyses were included in the calculations. Thus, the incorrect results resulting from technical errors were eliminated. Base peak chromatograms (BPCs) from each method displayed similar overall profiles (data not shown or available as Supporting Information). However, upon closer examination, a notable intensity increase was observed during the last 15 min of the 40 min gradient, specifically in Protocol A (Figure 3).

This increased intensity in Protocol A suggests that more hydrophobic peptides eluted during the final segment of the gradient. Given that the same gradient was used for all three protocols, the differences observed can be attributed to the variations in the protein content extracted by each protocol. Reversed-phase high-performance liquid chromatography (RP-HPLC), known for its effectiveness in separating peptides based on hydrophobic interactions, indicates that Protocol A is more efficient in extracting hydrophobic proteins (Figure 3).

**Figure 4:**
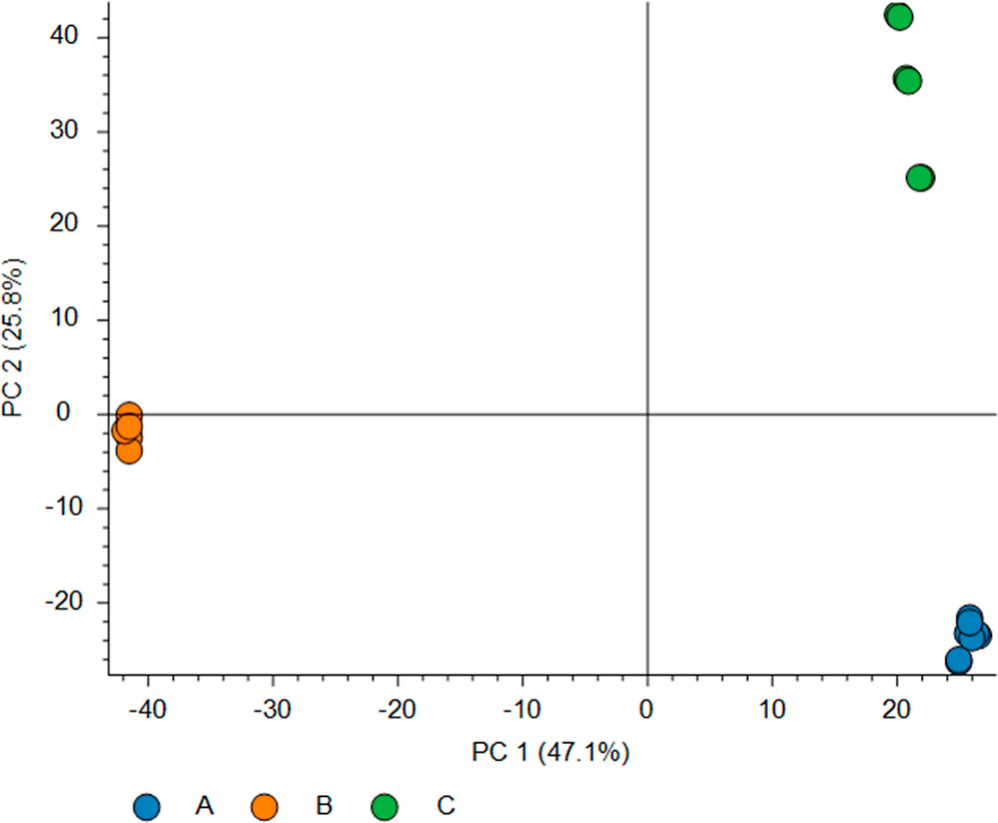
PCA plot based on normalized protein abundances obtained using different extraction protocols. Blue: Protocol A, orange: Protocol B, and green: Protocol C.

To further understand the differences in protein extraction efficiency among the protocols, a principal component analysis (PCA) was conducted based on normalized protein abundances (Figure 4). The PCA plot revealed three distinct clusters corresponding to the three extraction protocols. These sharp clusters indicate significant differences in the protein profiles obtained from each protocol.

**Figure 5:**
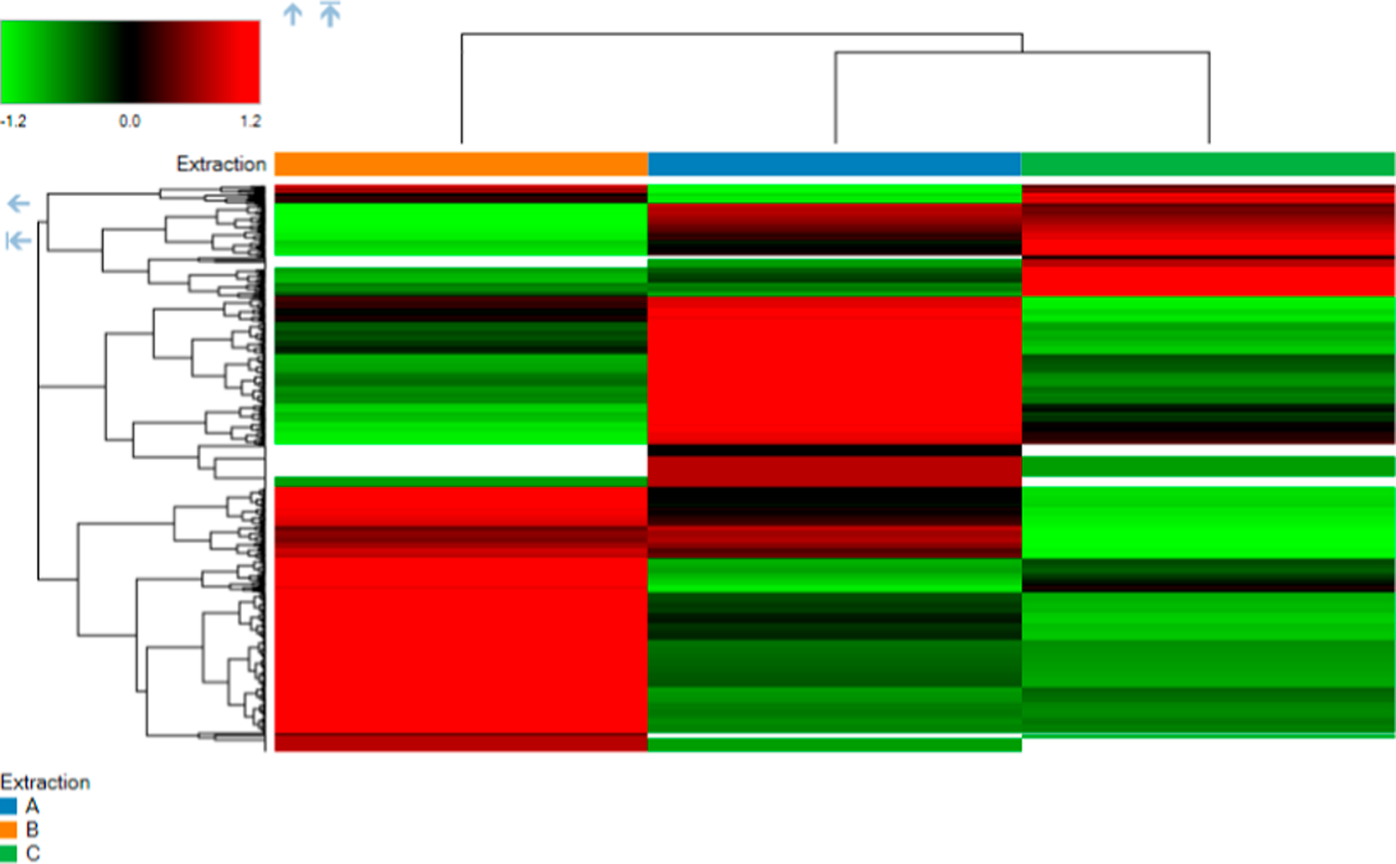
Heatmap showing differentially expressed proteins in three extraction protocols A, B, and C. The scale represents the log2 of protein abundance, ranging from low (green) to high (red) protein abundance. Gray shading indicates that the protein was not detected. The data in the heatmap represent three replicates for each extraction condition.

Further supporting these findings, the heatmap analysis displays the differentially expressed proteins across the three extraction protocols (Figure 5). The heatmap, which shows the log2 of protein abundance ranging from low (green) to high (red), highlights significant differences in protein expression. It allows for a clear visual comparison of protein expression across the three protocols. In Protocol A, there are a substantial number of red regions compared to those in Protocols B and C, indicating a higher abundance of individual proteins. Figure 5 demonstrates that Protocol A is particularly effective at extracting proteins across a wide range of abundances, especially those with higher expression levels. In contrast, Protocol C predominantly displays green regions, indicating lower protein abundances compared to those of Protocols A and B. The results indicate that Protocol A is the most effective at extracting a broader range of proteins. Although Protocol B also shows a red region, its range is narrower than that of Protocol A, suggesting that it extracts certain proteins effectively, but in lower amounts. In contrast, Protocol C predominantly displays green regions, indicating lower protein abundances compared to Protocols A and B (Figure 5).

**Figure 6:**
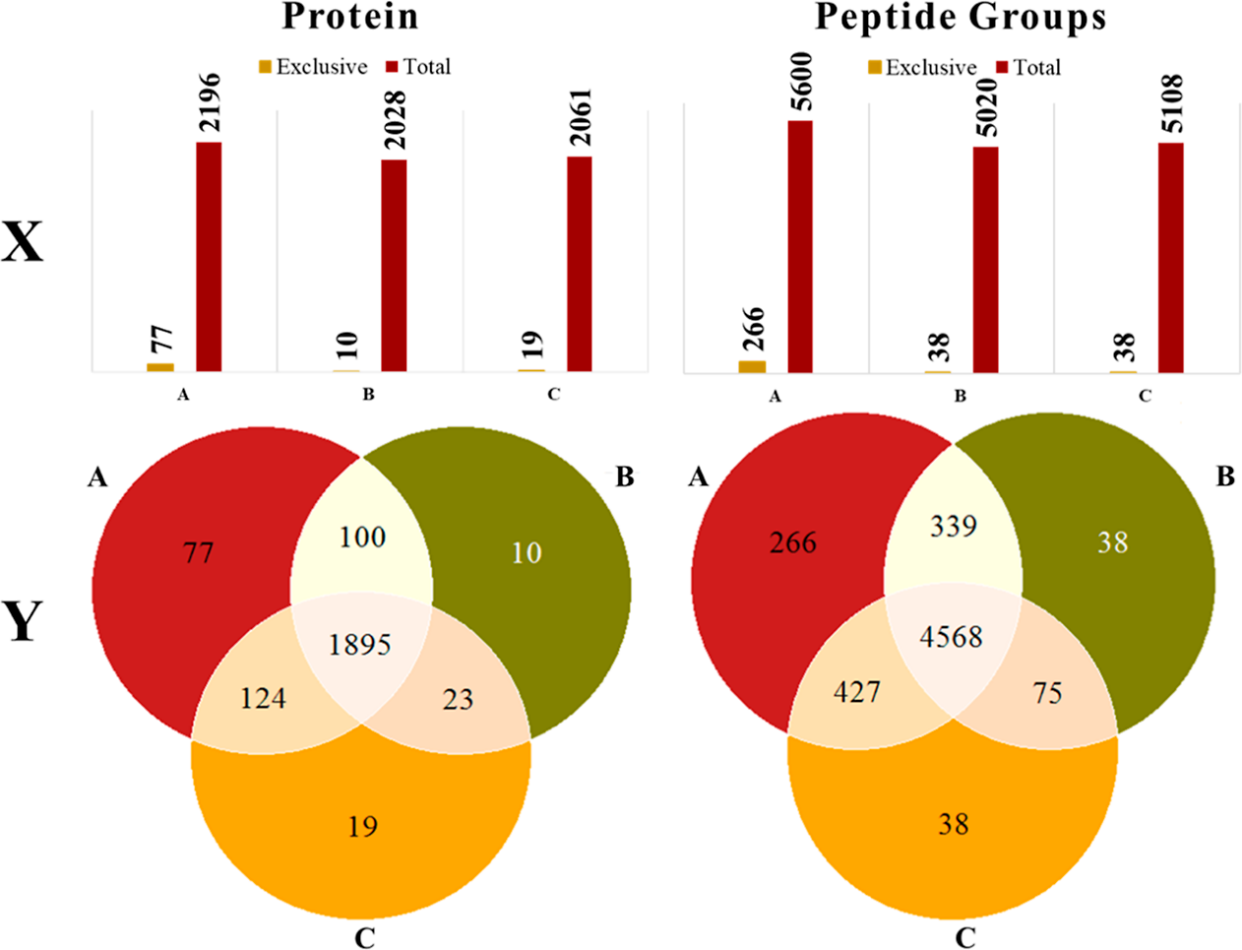
. Venn diagrams and bar chart representing the protein profiles obtained using three extraction protocols: proteins and peptide sequences. X (bar chart), Y (Venn diagram).

To further elucidate the unique protein and peptide profiles obtained by using the different extraction protocols, Venn diagrams were constructed (Figure 6). The Venn diagrams illustrate the overlap and exclusivity of proteins (A) and peptide sequences (B) identified in each protocol. Protocol A uniquely identified 77 proteins, while Protocols B and C identified 10 and 19 unique proteins, respectively. This suggests that Protocol A has a broader reach in extracting diverse protein species compared to Protocols B and C. Similarly, the peptide group analysis showed that Protocol A uniquely identified 266 peptide groups, while Protocols B and C each identified 38. This significant difference in the number of unique peptides detected further supports the notion that Protocol A is more efficient in extracting and preserving a comprehensive set of peptide groups, which is critical for in-depth proteomic analysis.

Plant tissues’ complexity, characterized by the abundance of phenolic compounds, presents persistent challenges in proteomic studies. Phenolic compounds can form covalent bonds with proteins, altering their structure and solubility The surface hydrophobicity, free sulfhydryl groups content, particle size, and zeta potential of protein isolates were significantly influenced by the formation of the phenol protein complex.^28^ Consequently, protein extraction becomes a critical step that significantly influences the quality and quantity of the proteomic data. Studies on *Spirodela polyrhiza* (duckweed)^29^ and liverworts^30^ have demonstrated the importance of optimized extraction methods in overcoming the challenges posed by plant tissues rich in phenolic compounds and secondary metabolites. For instance, in 2-DE studies, extraction with 50 mM tris-HCl (pH 7.5), followed by 20% TCA–acetone precipitation, was reported to be more effective for liverworts.^30^ Conversely, the trichloroacetic acid (TCA)/acetone/TCA precipitation method (TAT) provided better coverage and abundance of protein spots in duckweed.^29^ Our study’s success in addressing these challenges through Protocol A’s optimized conditions aligns with strategies proposed in the literature, such as the use of suitable solvents and buffers.^31^ Protocol A’s effectiveness in our study—particularly in extracting proteins with high hydrophobicity and broad isoelectric point (pI) ranges—resonates with findings that emphasize the need for modified extraction protocols. Phenolic compounds and other secondary metabolites, often coextracted with proteins, pose significant challenges by interfering with downstream analyses such as LC–MS/MS. Protocol A’s ability to minimize these interferences highlights the importance of using buffers and conditions that stabilize proteins while reducing contamination. This approach is consistent with advancements reported by Yan et al. (2020), exploring the impact of protein-phenolic complexes on protein solubility and functionality.^28^

The optimized extraction protocol in our study significantly improved the LC–MS/MS performance, as evidenced by higher peptide yields and enhanced chromatographic resolution. These results align with contemporary research on plant protein extraction, which emphasizes the importance of advanced methods such as TCA/acetone precipitation for preserving protein integrity.^31^ PCA and heatmap analyses further validated the utility of Protocol A in generating distinct proteomic profiles, enabling the identification of unique protein markers.

**Figure 7:**
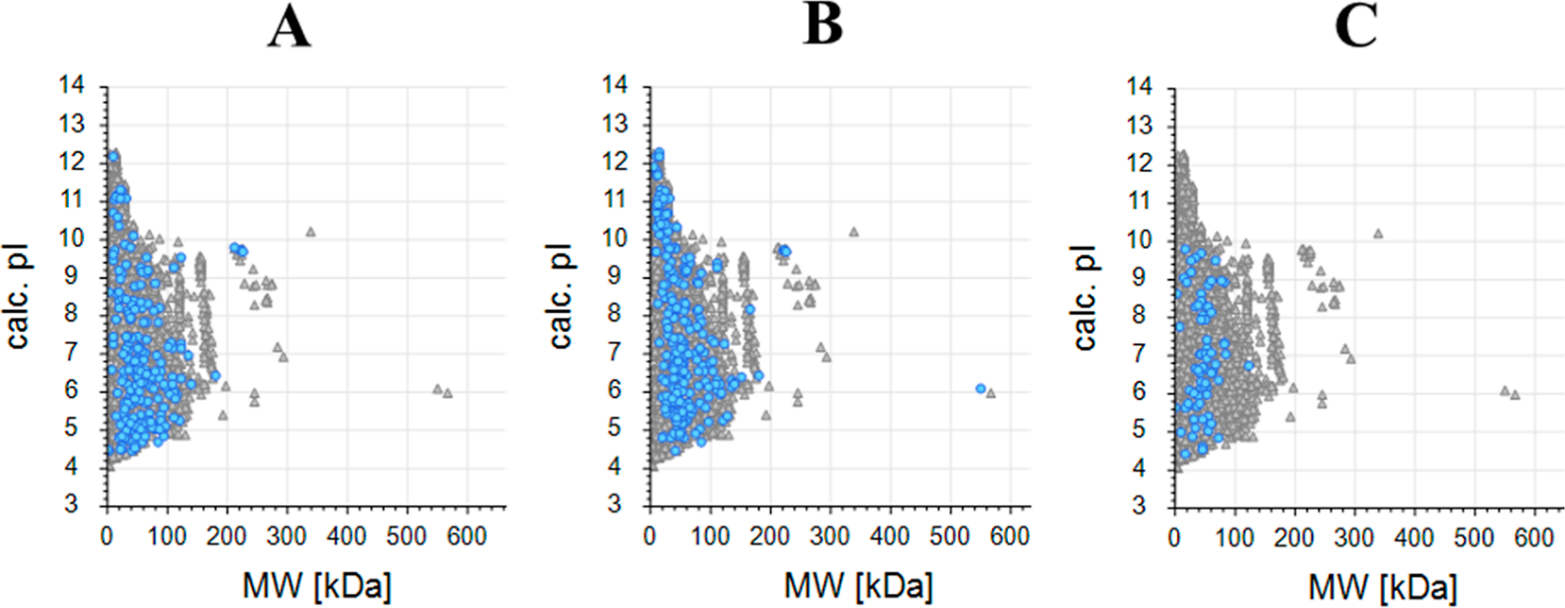
Scatter plots representing the MW and pI of differentially expressed proteins based on protein abundances. (A) 245 proteins with more than 2-fold abundance in Protocol A compared to that in Protocol B (log2 FC: 1, *P* < 0.05, FDR < 1.0%); (B) 216 proteins with more than 2-fold abundance in Protocol A compared to that in Protocol C; (C) 75 proteins with more than 2-fold abundance in Protocol B compared to that in Protocol C. Blue dots represent differentially expressed proteins, while gray dots show the distribution of all proteins.

Furthermore, the scatter plots in Figure 7 provide additional insights into the molecular weight (MW) and pI distributions of differentially expressed proteins among the protocols. Specifically, 245 proteins with more than 2-fold abundance in Protocol A compared to that in Protocol B (log2 FC: 1, *P* < 0.05, FDR < 1.0%) are represented in Figure 6A. Similarly, Figure 6B shows 216 proteins with more than 2-fold abundance in Protocol A compared to that in Protocol C, and Figure 6C highlights 75 proteins with more than 2-fold abundance in Protocol B compared to that in Protocol C.

The scatter plots reveal that despite the higher number of differentially abundant proteins in Protocol A compared to those in Protocols B and C, there is no significant clustering based on MW or pI. This indicates that the differentially abundant proteins extracted by Protocol A are distributed across a wide range of MW and pI values without a particular concentration in any specific range.

Similarly, the differentially abundant proteins in Protocol B compared with Protocol C also show a wide distribution in MW and pI values, suggesting that the extraction protocols do not favor proteins with specific MW or pI characteristics. This lack of significant clustering or concentration in the scatter plots highlights that the observed differences in protein abundances are not biased toward proteins of a particular size or charge but rather reflect a broad and diverse proteomic profile.

**Figure 8:**
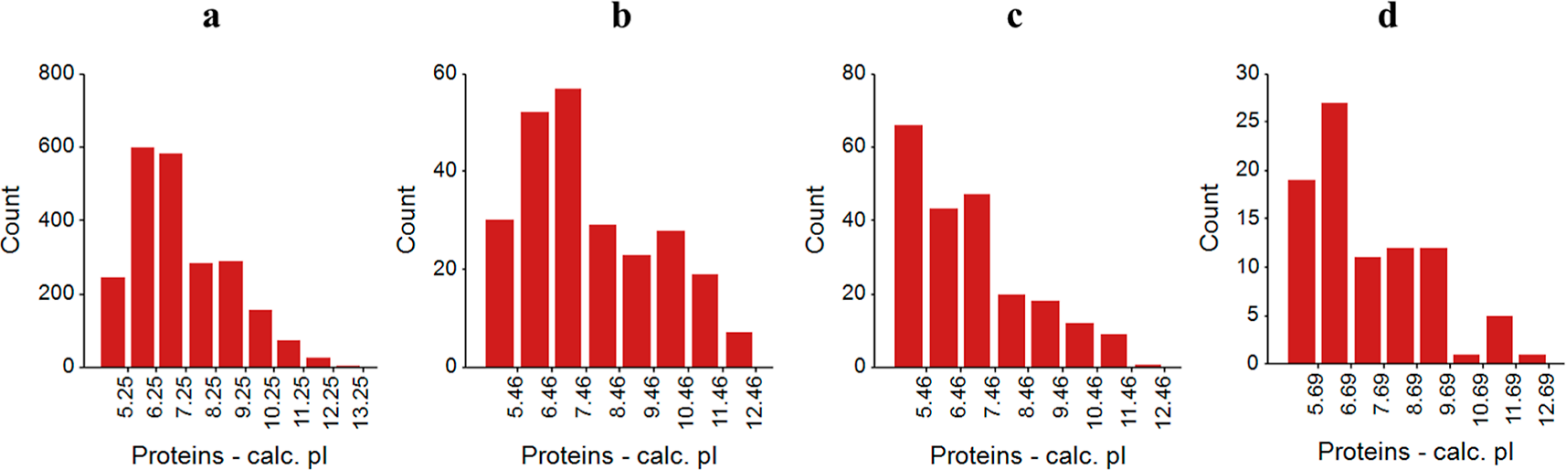
Histograms representing the distribution of pIs of differentially expressed proteins. (a) Distribution of all proteins; (b) proteins with increased abundance in Protocol A compared to that in Protocol B; (c) proteins with increased abundance in Protocol A compared to that in Protocol C; and (d) proteins exclusive to Protocol A.

Additionally, the histograms in Figure 8 represent the distribution of pIs of the differentially expressed proteins. The pI of a protein is the pH at which the protein carries no net charge.^32^ Proteins with different pI values behave differently under varying pH conditions, affecting their solubility and extraction during proteomics protocols. Acidic proteins (lower pI) and basic proteins (higher pI) require different conditions for effective extraction, which is why the effectiveness of different protocols can vary significantly across the pI spectrum.^33^

In Figure 8, Panel A shows the distribution of all proteins, providing a baseline for comparison. Panel B illustrates the pI distribution of proteins with increased abundance in Protocol A compared with that in Protocol B. Here, a significant number of proteins with pI values above 9.5 were observed in Protocol A, indicating an enhanced extraction of basic proteins. In Protocol A, a greater abundance of proteins with pI values between 4.5 and 6.5 was observed. This indicates Protocol A is also effective in extracting acidic proteins, which are often more easily solubilized due to their negative charge in physiological buffers.^34^ The enrichment of proteins with pI values above 9.5 in Protocol A compared to Protocol B observation aligns with findings that basic proteins are more challenging to extract due to their higher pI values, which can lead to poor solubility under standard conditions^35^· Protocol A’s effectiveness in extracting the basic proteins suggests that it employs conditions (e.g., higher pH or specific detergents) that stabilize these proteins during extraction, which is consistent with other research indicating the need for optimized buffers to handle high-pI proteins.^36^

In Figure 8, Panel C shows the pI distribution of proteins with increased abundance in Protocol A compared to that in Protocol C, where a higher abundance of proteins with pI values between 4.5 and 6.5 was observed in Protocol A. This suggests that Protocol A is also effective in extracting acidic proteins that are less abundant in Protocol C. Figure 8 Panel d presents the pI distribution of proteins exclusive to Protocol A. When compared to the total protein distribution, a noticeable increase in proteins with pI values between 4.5 and 7, as well as between 10.5 and 11.5, was observed. This indicates that Protocol A uniquely captures proteins across a broad pI range, including both acidic and basic proteins, enhancing the overall proteome coverage.

It was shown that proteins with lower pI values tend to be enriched in buffers with moderate to lower pH ranges, where they remain soluble.^37^ Thus, the effectiveness of Protocol A in this range implies that it optimizes conditions that allow for greater solubility of these acidic proteins compared to Protocol C.

The broader pI range covered by Protocol A indicates that this protocol enhances proteome coverage by effectively extracting proteins across the acidic and basic ends of the spectrum. This reflects the importance of using multiple conditions or optimized buffers to achieve more comprehensive proteome profiling.^38^ Studies show that single extraction protocols often lose proteins with extreme pI values (either very acidic or basic), but approaches that use altered conditions for different pI ranges can overcome this limitation.^39^ The findings emphasize the critical role of pI in determining the protein extraction efficiency. Protocol A demonstrates a wider pI coverage, enhancing its utility for comprehensive proteome analysis. This is consistent with proteomic studies emphasizing the importance of modifying extraction protocols to capture proteins across a diverse range of pIs to avoid biased proteome representation.

**Figure 9:**
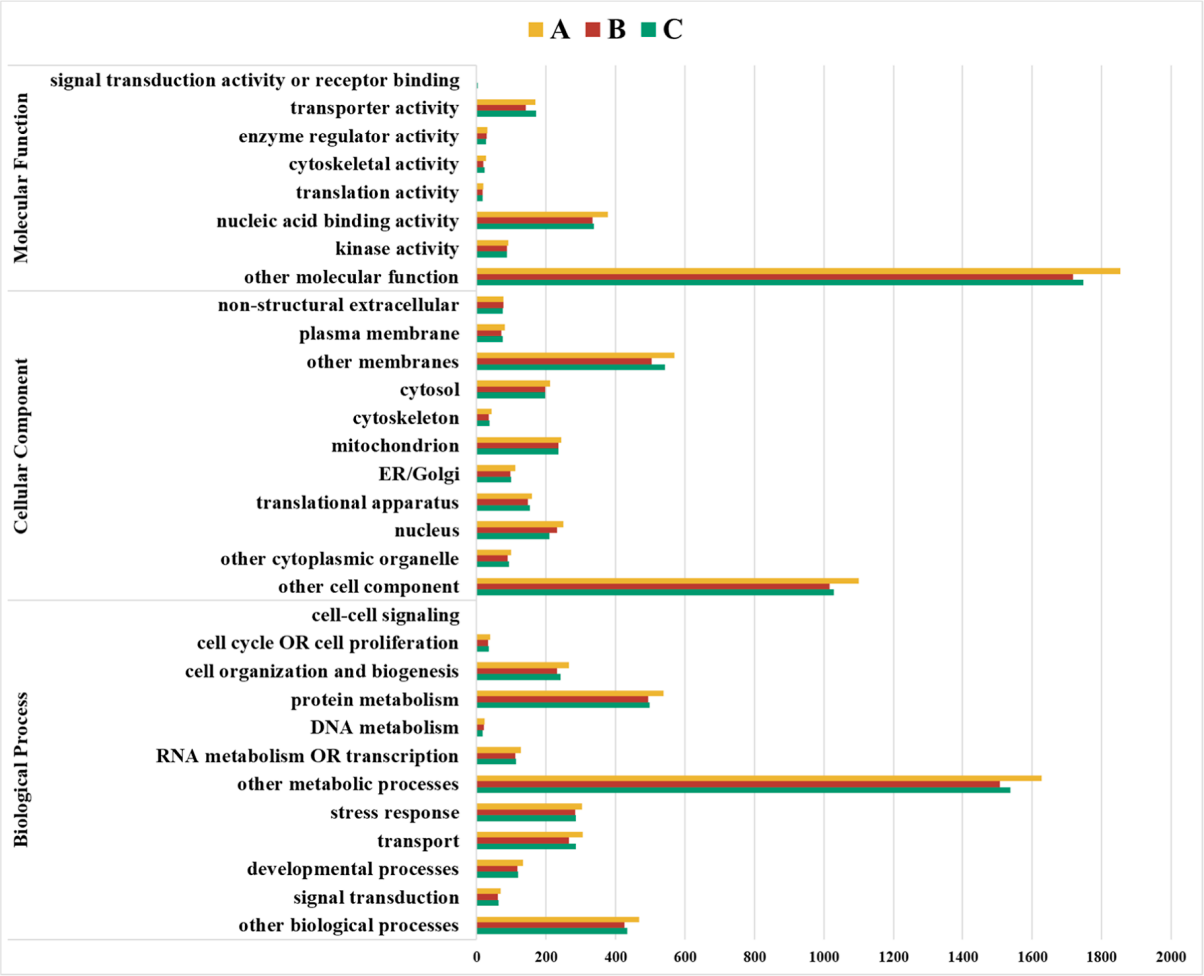
Bar charts displaying GO data for biological process, cellular component, and molecular function. A—SDS, B—CHAPS, and C—TCA/acetone.

The gene ontology (GO) database was used to categorize differentially expressed proteins enriched by three extraction methods. For this purpose, three main hierarchically structured GO terms were used: the biological process, the cellular components, and the molecular functions. Generally, the highest yield from the relevant protein groups within the molecular function pathways was obtained from Protocol A. Protocol C was more effective than Protocol A in detecting proteins in the “transporter activity” and “signal transduction activity or receptor binding” groups. Protocol B was found to be the least efficient protocol for identifying protein groups associated with molecular function pathways. It was determined that Protocol B was more efficient than Protocol C only in the “kinase activity” and “enzyme regulator activity” groups (Figure 9).

The challenges of extracting proteins from plant tissues abundant in secondary metabolites were addressed by incorporating conditions that effectively solubilize proteins while minimizing degradation. Studies on sago palm^27^ and *Cinnamomum camphora*(28) have similarly underscored the importance of optimizing extraction conditions to enhance proteome coverage and quality. Furthermore, the influence of phenolic and secondary metabolites on proteomic workflows highlights the need for methodological advancements. As emphasized by Yadav et al. (2020), removing these interfering compounds is essential for producing consistent proteomic data.^30^ Our results demonstrate that comprehensive optimization of extraction protocols can mitigate these issues, ensuring robust and reproducible results.

The study established a detailed protocol for the isolation and analysis of proteins from *O. europaea* leaves, applying a series of optimized extraction, quantification, and LC–MS/MS techniques. The results revealed that the selected methodologies provide a robust and reproducible basis for studying the proteome of olive leaves. The improvement in yield and purity of extracted protein has significant effects for downstream applications, such as proteomic studies exploring the abiotic and biotic stress responses of olive plants, which are essential for developing more tolerant/resistant cultivars in different environmental stresses. The identification of unique proteins suggests that the SDS Protocol (Protocol A) may lead to broader coverage of the olive proteome compared to other protocols. The increased proteomic depth can open new avenues for research, particularly in understanding the molecular mechanisms underlying olive plant productivity and sustainability.^40^

The characterization of proteins involved in lipid metabolism has potential applications in enhancing the nutritional quality of olive oil, which is a key product of the olive industry. By leveraging the proteomic data, future studies may focus on modulating protein expression to maximize oil quality specifications, particularly in terms of fatty acid profiles and antioxidant content, which are critical for both human nutrition and commercial value. The potential loss of low-abundance proteins during the extraction and purification steps may limit the detection of important regulatory proteins.^41^ Future optimizations could focus on improving these steps to minimize protein loss and increase the detection of these elusive proteins. However, while the study focused on olive leaves, application of the SDS protocol to other tissues, such as fruits or roots, may provide a more comprehensive understanding of the olive proteome. Expending the analysis to other tissues will also help comparisons between different developmental stages or environmental conditions and potentially reveal tissue-specific proteins that play important roles in cellular processes and their potential applications in olive breeding programs.^19^

Rigorous optimization of sample preparation, chromatographic conditions, and MS parameters customized to the specific characteristics of olive leaf extracts is important for accurate LC–MS/MS analysis. Collaboration among analytical chemists, molecular biologists, and bioinformaticians can help overcome these difficulties and achieve successful LC–MS/MS analysis of proteins from olive leaves.

## Data Availability

All data generated or analyzed during this study are included in this published article.

## Supporting Information Available

LC–MS base peak chromatograms of peptides obtained by Extraction Protocol A, Extraction Protocol B, and Extraction Protocol C (PDF)
