# Supplementary material for: Comparative Analysis of Protein Extraction Protocols for Olive Leaf Proteomics: Insights into Differential Protein Abundance and Isoelectric Point Distribution
Source: ACS Agric Sci Technol. 2025 Mar 13;5(5):739–49. doi: 10.1021/acsagscitech.4c00642 (PMC12093291; doi:10.1021/acsagscitech.4c00642)
Supplement: Supplementary file 1 — as4c00642_si_001.pdf [file as4c00642_si_001.pdf]

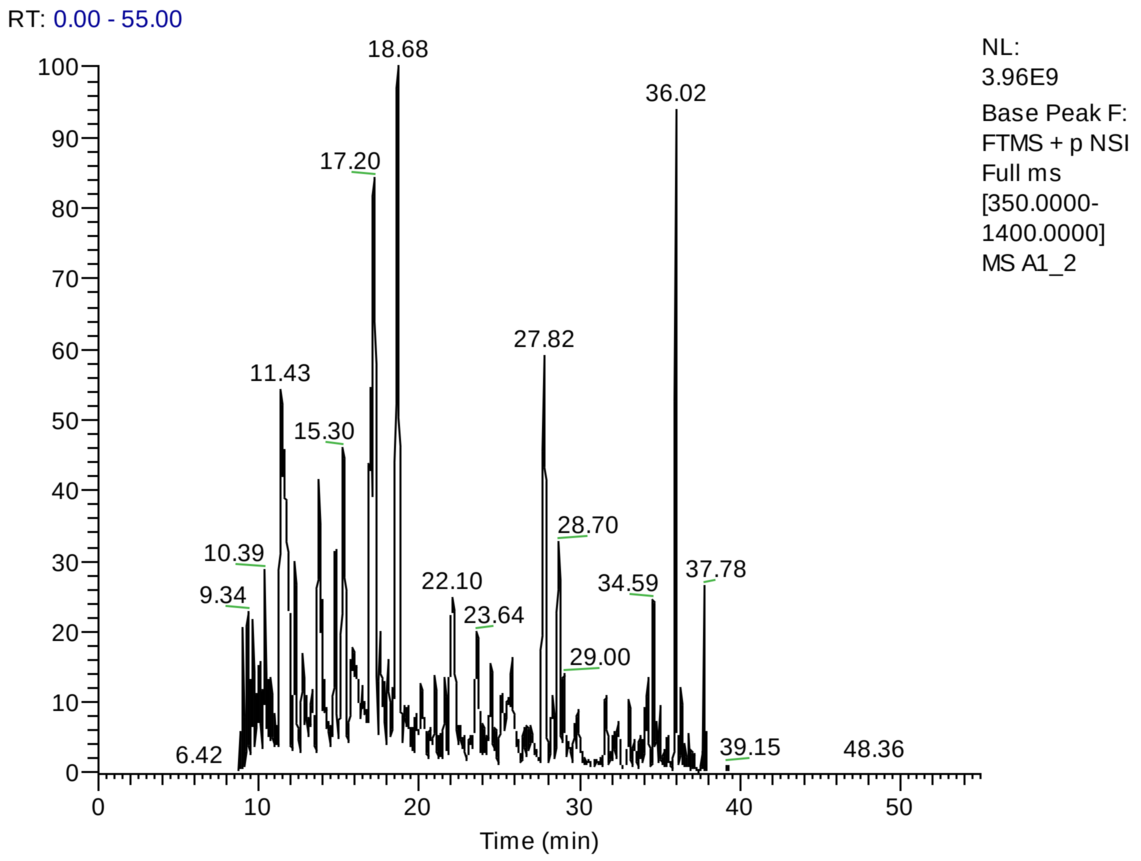

Suplementary Figure 1: LC-MS Base Peak Chromatograms of Peptides Obtained by Extraction Protocol A

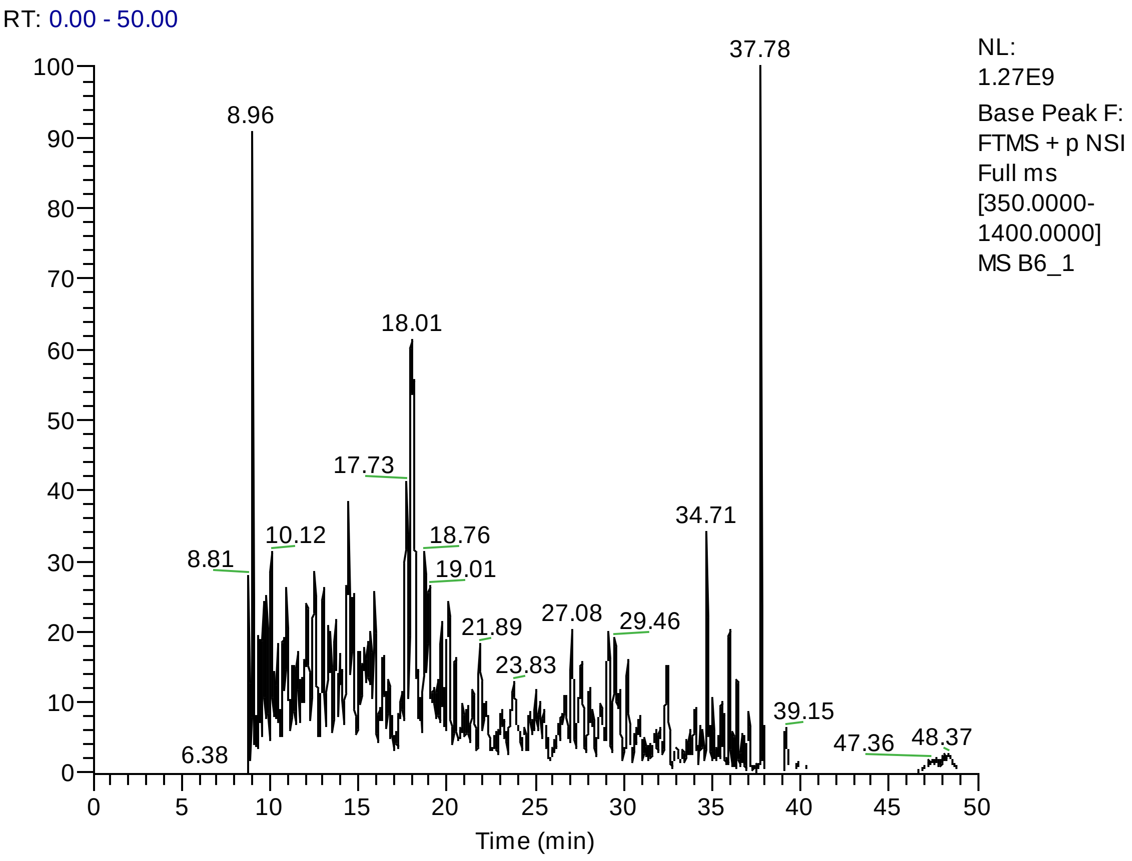

Suplementary Figure 2: LC-MS Base Peak Chromatograms of Peptides Obtained by Extraction Protocol B

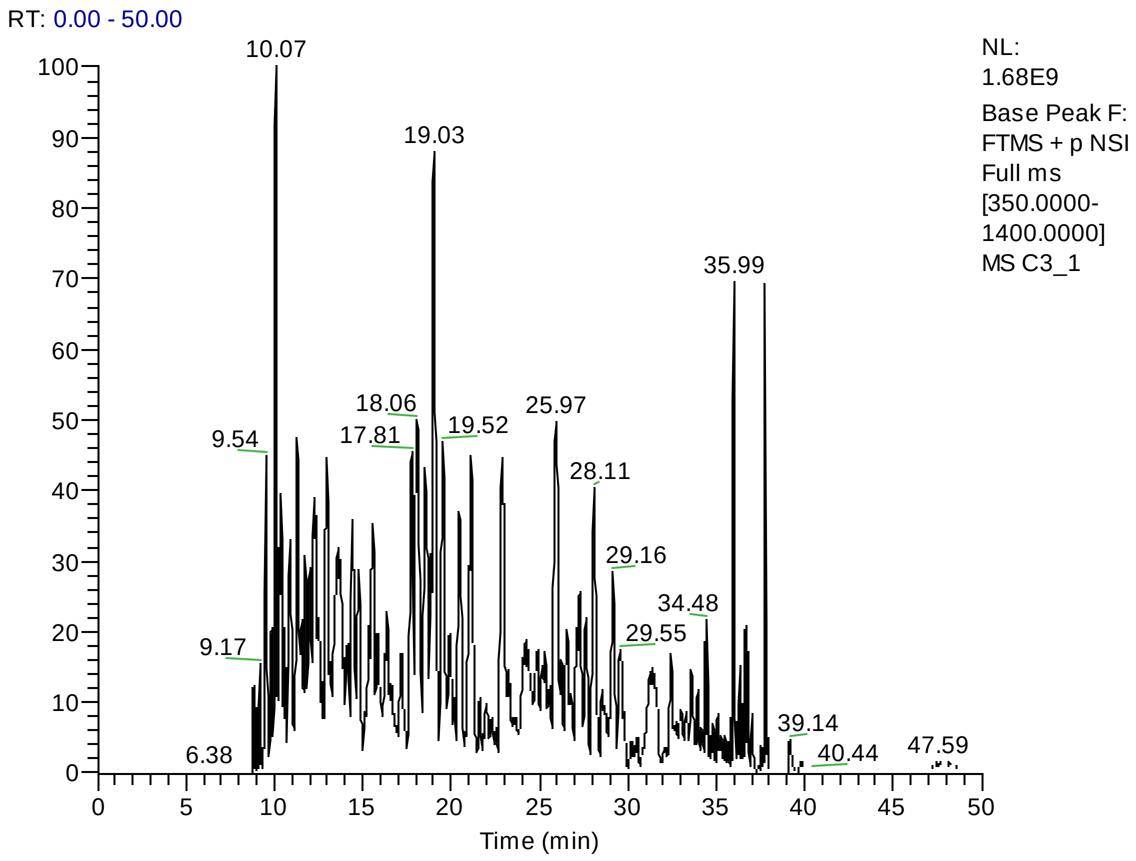

Suplementary Figure 3: LC-MS Base Peak Chromatograms of Peptides Obtained by Extraction Protocol C
